# Developmental trajectories of eye movements in oral and silent reading for beginning readers: a longitudinal investigation

**DOI:** 10.1038/s41598-022-23420-5

**Published:** 2022-11-04

**Authors:** Young-Suk Grace Kim, Callie Little, Yaacov Petscher, Christian Vorstius

**Affiliations:** 1 School of Education, University of California Irvine, 3200 Education Building, Office 3225, Irvine, CA 92697 USA; 2 Florida State University, Tallahassee, USA; 3 University of Wuppertal, Wuppertal, Germany

**Keywords:** Neuroscience, Psychology

## Abstract

Eye movements provide a sensitive window into cognitive processing during reading. In the present study, we investigated beginning readers’ longitudinal changes in temporal and spatial measures of eye movements during oral versus silent reading, the extent to which variation in eye movements is attributable to individual differences and text differences, and the functional form of growth trajectories of eye-movement variables. Data were from 363 English-speaking children (52% male; 59.8% White) in the US who were followed longitudinally from Grade 1 to Grade 3. Results showed a rapid decrease in temporal eye-movement measures (e.g., first fixation) and an increase in spatial eye-movement measures (initial landing position) in both oral and silent reading. The majority of variance in eye movements was attributable to individual differences whereas some variance in initial landing position was due to text differences. Most eye-movement measures had nonlinear growth trajectories where fast development tapered off near the end of Grade 3 while initial fixation count and total gaze count in silent reading had a linear growth trajectory. The findings provide a first large-scale look into the developmental progression of eye movements during oral and silent reading during a critical period when reading skills rapidly develop.

## Introduction

Reading is a developmental process. Numerous studies have shown that beginning readers assemble phonological, orthographic, and semantic information in a slow, laborious manner. As reading skills develop, individuals build automaticity in word reading to support deep understanding of written texts. However, little is known about developmental changes and trajectories of underlying cognitive processes during reading^1^. Eye movements provide a useful, sensitive window into cognitive processing^2–4^ and, therefore, are ideal for examining developmental changes in cognitive processes^1^. In the present study, we examined growth trajectories of text reading processes captured by eye movements using large-scale longitudinal data from the beginning of Grade 1 to end of Grade 3, a period during which reading skills develop rapidly. Furthermore, we investigated the extent to which variation in children’s eye movements during text reading is attributable to individual differences and text differences.

### Eye movements during reading

Eye-movement behaviors are associated with specific underlying cognitive processes (see computational reading models^5–7^). While differing in assumptions regarding the extent of parallel versus serial processing, computational models like SWIFT^5^, Glenmore^7^, and E-Z reader^6^, hypothesize that eye movements are primarily driven by word reading and associated orthographic, phonological, and semantic processes as well as attention and oculomotor control. Several temporal and spatial eye-movement measures are widely used to capture underlying cognitive processes during reading. Temporal measures capture how much time is spent processing information whereas spatial measures capture where information is extracted^3^. Temporal measures include amount of time spent looking at a word (e.g., initial fixation duration, rereading duration) and count/proportion looking at a word (e.g., number of initial fixations and gazes, number of total fixations). Spatial measures include saccade amplitude (how far the eyes move in a saccade) and initial landing position (where within a word eyes land initially). Initial fixation duration—the amount of time spent on a word at first fixation—is hypothesized to capture initial decoding processes. Refixation duration—the amount of time spent refixating a word after initial fixation—is hypothesized to capture lexical access^8–10^, and rereading duration—coming back to the word after having read one or multiple other words—is posited to capture higher order syntactic integration processes^11–14^.

Proficient adult readers typically read in silent mode, and they spend approximately 200–250 ms, on average, during initial fixation on a word, an additional 100–120 ms when refixating on the same word (refixation duration), and approximately 200–220 ms when rereading the same word (rereading duration^3,12^). When it comes to spatial measures, proficient adult readers’ saccades, again in silent reading, are six to eight letters, and the initial landing position is about halfway between the beginning and the middle of a word^15,16^.

### Eye movements for beginning readers and growth of eye-movement measures

The vast majority of previous work on eye movements has been conducted with adult readers. However, a growing number of recent studies have examined eye movements for beginning readers and children^1,17–23^. One key difference between typical adults and children is their reading proficiency. Reading proficiency influences readers’ eye movements^17–21,24^. Studies have shown similarities and differences between adult readers and developing readers. Like adult readers, developing readers manifest shorter fixation duration for high-frequency words than for low-frequency words. However, the perceptual span, the area from which necessary information can be extracted (e.g., word boundary or letter identity), is smaller for beginning and poor readers at least until the age of 11–12 years, which results in extraction of less information from the parafovea^21,25,26^, the area around the current fixation location.

As children develop their reading skills, their eye movements should change over time because eye movements reflect reading skills and underlying cognitive processes (see above). Indeed, studies have confirmed changes in eye movements with reading development. In their cross-sectional study with students in Grades 1 to 5, Vorstius and colleagues showed that time spent fixating on words (initial fixation duration, refixation, and rereading duration) and the number/proportion of rereading words decreased from Grade 1 to Grade 4 while these eye-movement measures stabilized in Grades 4 and 5^22^. A one-year longitudinal study with German-speaking children from Grade 1 to 3 (i.e., Grade 1 followed to Grade 2; Grade 2 followed to Grade 3) found that changes in the perceptual span were greater from Grade 2 to Grade 3 than from Grade 1 to Grade 2^21^. McConkie and colleagues (1991) used 12 children’s data from Grade 1 to Grade 5 and reported that fixation durations decreased whereas percent regressions and saccade length increased^27^. Kim and colleagues followed students from the beginning to the end of the year in Grade 1 and found decreases in temporal measures (initial fixation duration, refixation duration, rereading time, number of fixations in gaze)^17^.

These findings of changes in eye movements as children develop reading skills are in line with the phases of reading development (e.g., see a model of word reading development^28^). In the very initial phase of reading development, decoding or word reading develops from slow and laborious small-unit based reading (e.g., letter-by-letter reading), which requires longer and more frequent fixation duration (initial and refixation time). As children develop reading skills, their word reading becomes automatized, reducing fixation duration and frequency^15,17,22^.

However, to our knowledge, no prior study examined growth trajectories of eye-movement parameters. An exception is the De Luca and colleagues’ study which measured eye movements of an Italian-speaking girl from Grade 1 to end of Grade 5. The study found a rapid change in eye movements (fixation duration and count) in Grade 1 followed by substantial slowdown in changes afterwards^29^.

### Oral versus silent reading for children

Proficient readers typically adopt silent reading as the primary mode of reading, and they read faster in silent than oral mode^3^. Oral reading involves more ongoing processes than silent reading because processes related to overt language production, such as sounding out words and prosody, have to be integrated with ongoing reading. Perhaps not surprisingly, oral versus silent reading mode influences temporal and spatial measures of eye movement^20,23,30,31^. Oral versus silent reading is particularly relevant to developing readers as they start in oral reading mode and transition to silent reading^17^. However, only a few studies investigated oral versus silent reading for developing readers. Vorstius and colleagues examined English-speaking students in Grades 1 to 5 for their sentence reading in oral and silent mode and found longer fixation duration, higher fixation count, and smaller saccade amplitude in oral reading than in silent reading^22^. A study with German-speaking adolescents (mean age = 13 years and 6 months) also showed that oral reading has longer total reading time, word reading time, initial fixation duration, refixation duration, and rereading duration; higher fixation count and number of saccades; and shorter saccade amplitude^32^. It is likely that fixation duration is longer and the number of fixations is higher in oral reading mode because the probability of fixating every word is higher and initial landing position is shorter in oral reading which is less optimal for word reading and leads to refixations.

Our understanding of *developmental trajectories* of oral versus silent reading is extremely limited. One study worked with a cross-sectional sample of English-speaking students in Grades 1 to 5 and found that differences in oral reading and silent reading persisted, but the pattern of decrease in temporal measures (e.g., initial fixation duration, refixation duration) was similar in oral and silent reading modes^22^. In another study, students in Grade 1 were followed from fall to spring and their temporal measures of eye movements decreased. Interestingly, the extent of decrease was consistently larger in oral reading mode than silent reading mode^17^. Overall, these studies indicate that readers fixate longer and more frequently in oral reading mode, and fixation duration and frequency/proportion in oral and silent reading mode decrease with reading development.

### Individual differences and text differences

A large body of studies has consistently shown large variation in development of reading skills among children, and a large proportion of children’s performance on reading tasks is a function of students’ reading skills^33^. Therefore, a large proportion of differences in eye movements should be attributable to between-child differences.

On the other hand, another line of work also clearly showed the roles of word and text features in reading. Words differ in several aspects such as length, frequency, familiarity, and consistency in letter-sound correspondences, and these differences influence one’s word reading and associated eye movements. Specifically, familiar words, shorter words, and frequently occurring words are fixated for a shorter time^6,8,34,35^ and are skipped more often^36^. Beyond word features, texts also differ, including in language and content demands, cohesion, coherence, and structure^37–39^, and differences in texts explain performance differences in reading^33^. If word and text features influence one’s reading performance, it is reasonable then to hypothesize that at least some of the variation in eye movements is attributable to differences in texts. To our knowledge, the extent to which variability in eye movements is attributable to between-individual differences and between-text differences has not been examined in prior studies of eye movements.

### Present study

In this study, we examined developmental changes and growth trajectories of eye-movement measures for English-speaking students in primary grades, during which children are experiencing rapid development of reading skills. Although previous studies were highly informative, longitudinal studies of eye-movement development are extremely scarce and to our knowledge, no prior work has estimated functional forms of growth trajectories of eye-movement behaviors over time. Furthermore, little systematic information is available about developmental trajectories of eye-movement measures in oral versus silent reading. Also absent is information about the extent to which variances in eye movements are attributable to individual differences and text differences. In the present study, we addressed these gaps in the literature guided by the following research questions.

1. What are developmental changes in eye movements during text reading in oral and silent reading modes from Grade 1 to Grade 3 for English-speaking children?
2. To what extent is variability in eye movements attributable to between-child differences and text differences in oral and silent reading?
3. What functional form of growth trajectories best characterizes eye movements during text reading from Grade 1 to Grade 3 for English-speaking children? Are growth trajectories similar or different in oral versus silent reading mode?

These questions were addressed using a large-scale longitudinal data set from Grade 1 to Grade 3. Given rapid development of reading during primary grades, eye movements were measured twice within an academic year (in the fall and spring of each year) for a total of six waves of data.

## Method

### Participants

The sample was composed of 363 English-speaking children (52% male) from seven schools in two school districts in a southeastern state in the US. These students were followed longitudinally and assessed in the fall and spring of Grade 1 (fall mean age = 6.36 years [*SD* = 0.53]), Grade 2 (fall mean age = 7.33 years [*SD* = 0.52]), and Grade 3 (fall mean age = 8.34 years [*SD* = 0.54]). The racial/ethnic breakdown was as follows in Grade 1: 59.8% White children, 25.9% African American children, 5.9% Hispanic children, 2.4% Asian/Pacific Islander children, and 5.9% identified as two or more races/ethnicities. Approximately 52% of students in Grade 1 (*n* = 192), 46% of students in Grade 2 (*n* = 172), and 39% of students in Grade 3 (*n* = 146) were eligible for the free or reduced lunch program, a proxy for low-income status. The school district record showed only a small number of students (*n* = 3) with limited English proficiency in Grade 1. The study was approved by the Florida State University’s Institutional Review Borad (HSC NO. 2015.16488), and informed contents were obtained from participating children’s parents or their legal guardians. We confirm that all methods were performed in accordance with relevant guidelines and regulations.

Based on these criteria, analyses were based on *n*_*silent*_ = 262 and *n*_*oral*_ = 282 in Wave 1, *n*_*silent*_ = 338 and *n*_*oral*_ = 345 in Wave 2, *n*_*silent*_ = 322 and *n*_*oral*_ = 323 in Wave 3, *n*_*silent*_ = 319 and *n*_*oral*_ = 322 in Wave 4, *n*_*silent*_ = 306 and *n*_*oral*_ = 301 in Wave 5, and *n*_*silent*_ = 290 and *n*_*oral*_ = 292 in Wave 6. There were no missing data on eye-tracking measures within time points, but sample sizes across the time points indicated a smaller sample at Wave 1 than for subsequent time points with some attrition (14%) occurring between Wave 2 and Wave 6. Attrition bias was examined through several socio-demographic variables: age at Wave 1, gender, free and reduced-price lunch status, limited English proficiency, Year 1 primary exceptionality, and race/ethnicity. Mean comparisons across waves indicated no evidence of differential attrition across Waves 2 through 6.

### Measures

#### Eye movements during text reading

Three grade-level passages were presented to children at each of the six assessment points. The passages were equated and normed in the State of Florida, US. Grade 1 passages had 155–198 words (400–700 Lexiles), Grade 2 passages had 187–200 words (600–780 Lexiles), and Grade 3 passages had 200–307 words (400–790 Lexiles). One passage at each time point served as the linking passage between time points (i.e., one passage at Wave 2 was also given at Wave 3, etc.) and thus a total of 13 different passages were used across the six time points. The passages were composed of narrative and expository texts (6 out of 13 passages in the present study were narratives). Parallel form reliability exceeded 0.90 across the three passages within each time point. Test–retest reliability ranged from 0.87–0.88 for linking passages.

The same passages were used for both oral reading and silent reading sessions within each assessment wave. The order of oral and silent reading sessions, which were approximately one week apart within each assessment time point, was counterbalanced across children. Passages were presented on a computer monitor, and children were asked to read the texts aloud in the oral reading session and silently in the silent reading session. To ensure that children read the passages for meaning rather than for speed, one literal comprehension question (correct answer is explicitly provided in the given text; e.g., name of a character in the passage) was asked after each passage and answers were digitally recorded using digital recorders such as Olympus VN 8100 pc. These questions were designed for a manipulation check and were not expected to be a reliable measure of comprehension. Therefore, children’s performance on these comprehension questions was not used in the analysis.

Children’s eye movements were captured by an unobtrusive desktop camera in front of the monitor using the EyeLink1000 system in combination with a forehead and chin rest. Texts were presented on a 21-inch monitor with a screen resolution of 1024*768 pixels. Courier New typeface in 15-point font size was used, and viewing distance was adjusted so that one letter corresponded to 0.33 degree of visual angle. Texts were presented in black color on a grey background with double line spacing. Passages were broken up into two to three paragraphs, each paragraph consisting of five to seven lines and presented on a separate screen. Children were encouraged to move as little as possible during the measurement but could move around between passages. Between passages, the camera was calibrated to ensure measurement accuracy using a 9-point calibration and validation. Then, right before the child read a paragraph, an additional drift correction check was performed. If deviations larger than 0.5 degree of visual angle were detected, the camera system was recalibrated. Eye movements were tracked at 500 Hz, and viewing and recording were binocular, though only data from the right eye were used for analyses. Eye-movement data were processed and analyzed using EyeMap^40^ and SPSS. From the eye-movement data, eight measures were used in the analysis: Initial Fixation Duration, Refixation Duration, Rereading Duration, Initial Fixation Count, Total Fixation Count, and Total Gaze Count were temporal measures; and Saccade Amplitude and Initial Landing Position were spatial measures.

To ensure that children were “readers,” an oral or silent reading session was discontinued if the child could not read a single word at all or exceeded 5 min on a passage. Moreover, in a silent reading session where the experimenter cannot hear reading, the session was discontinued if it was apparent to the experimenter that the child was not reading, based on erratic fixation patterns or if the child physically disengaged from the chin rest. In addition, data sets with less than 100 fixations per passage were excluded from analyses.

### Data analytic strategies

Linear mixed effect models (LMEMs) were used to model individual growth curves in each of the eight eye-movement measures. The complexity of the data structure necessitated a preliminary evaluation of random effects to evaluate the extent to which observed or latent random effects models would be more appropriate to the data. Specifically, students were administered three passages at each wave of data collection, and thus, eye-tracking scores were cross-classified by students and passages. As well, students were nested within schools. Unconditional LMEMs estimated the grand mean for each eye-movement measure as well as the variance component at each wave, for each of the oral or silent reading conditions, associated with between-student differences, between-passage differences, between-school differences, and residual effects. Intraclass correlations (ICCs) contextualized the variances as the proportion of variance due to each source of clustering. A key evaluative component of the ICCs was the extent to which variance in the eye-movement variables was due to passage effects. A moderate to large portion of variance in scores attributed to passage might necessitate additional analytic considerations in modeling (e.g., latent variable measurement models with longitudinal invariance) whereas smaller variances across outcomes may allow for aggregate index use. The latter approach is similar to processes used in curriculum-based measurement whereby multiple passages are administered and scored, yet the median (or mean) is used to represent student performance^41,42^.

Individual growth curve analyses were then employed to estimate the functional form of growth for each eye-movement measure by condition. The availability of six waves of data allowed for curvilinearity testing; our model calibration process included linear, quadratic, and cubic models using the deviance statistics Akaike Information Criteria (AIC) and Bayes Information Criteria (BIC) to select the most appropriate model. The work reported here was not preregistered.

## Results

### Research question 1: developmental changes in eye movements from grade 1 to grade 3

Descriptive statistics for eye-movement measures are reported in Table 1 by wave and oral versus silent reading mode. Mean values decreased from Wave 1 to Wave 6 for all measures with the exception of average of incoming saccade amplitude and average initial landing position, which showed increasing values over time. Mean differences between oral and silent reading modes were statistically significant for all measures across all waves with the exception of initial landing position in Wave 6 (Table 2). Furthermore, temporal measures were consistently larger in the oral reading condition than in the silent reading condition across the six time points or waves.

**Table 1:** Descriptive statistics among eye-tracking measures by wave and oral versus silent reading mode.

Wave	Measure	Oral reading						Silent reading					
		Mean	*SD*	Min	Max	Skew	*n*	Mean	*SD*	Min	Max	Skew	*n*
1	Initial fixation duration	376.30	79.25	230.27	655.96	0.78	282	351.24	75.84	189.42	610.44	0.70	262
	Refixation duration	333.66	201.43	20.32	1376.64	1.52	282	233.29	145.34	36.34	948.88	1.65	262
	Rereading duration	632.35	529.99	51.11	2869.68	1.53	282	343.83	309.45	20.05	2270.76	2.57	262
	Initial fixation count	1.82	0.35	1.12	3.10	1.00	282	1.62	0.29	1.13	3.18	1.40	262
	Total fixation count	3.42	1.41	1.35	9.80	1.46	282	2.56	0.89	1.30	7.15	1.79	262
	Total gaze count	1.91	0.61	1.20	5.38	2.16	282	1.59	0.37	1.07	3.50	1.84	262
	Saccade amplitude	1.89	0.63	1.02	7.61	3.71	282	2.37	0.98	1.01	6.57	1.51	262
	Initial landing position	1.74	0.25	0.97	2.61	0.09	282	1.91	0.27	1.24	2.87	0.20	262
2	Initial fixation duration	358.74	75.55	124.57	648.25	0.64	345	332.58	68.92	202.70	543.01	0.40	338
	Refixation duration	235.05	127.31	30.93	751.92	1.05	345	186.60	107.03	22.76	648.04	1.23	338
	Rereading duration	375.84	273.51	57.87	1680.99	1.61	345	259.52	213.55	5.73	1636.34	2.69	338
	Initial fixation count	1.65	0.27	1.14	2.92	1.06	345	1.55	0.24	1.11	2.48	0.79	338
	Total fixation count	2.71	0.84	1.42	6.64	1.34	345	2.32	0.67	1.20	5.52	1.52	338
	Total gaze count	1.68	0.36	1.18	3.78	1.64	345	1.51	0.29	1.03	2.97	1.85	338
	Saccade amplitude	1.84	0.42	0.96	3.35	0.77	345	2.28	0.86	0.94	6.98	1.93	338
	Initial landing position	1.78	0.20	1.17	2.45	− 0.02	345	1.86	0.23	1.23	2.48	0.03	338
3	Initial fixation duration	332.18	70.79	203.84	708.64	0.97	323	315.18	66.12	177.14	534.42	0.56	322
	Refixation duration	194.88	114.51	31.15	644.45	1.25	323	150.15	88.80	15.97	523.59	1.19	322
	Rereading duration	311.03	246.51	38.19	1480.79	2.18	323	211.78	175.71	21.29	1489.92	3.01	322
	Initial fixation count	1.57	0.25	1.10	2.94	1.37	323	1.47	0.22	1.08	2.55	1.24	322
	Total fixation count	2.50	0.87	1.43	10.40	3.44	323	2.13	0.58	1.19	5.00	1.74	322
	Total gaze count	1.61	0.34	1.11	3.49	2.33	323	1.46	0.26	1.06	2.81	1.85	322
	Saccade amplitude	2.11	0.53	1.18	4.45	1.12	323	2.45	0.83	1.12	5.75	1.48	322
	Initial landing position	1.95	0.24	1.38	2.61	0.07	323	2.09	0.25	1.43	2.94	0.14	322
4	Initial fixation duration	304.12	56.99	178.10	531.19	1.00	322	292.83	57.41	156.02	523.60	0.74	319
	Refixation duration	155.39	89.65	32.64	655.22	1.61	322	124.64	85.53	9.10	667.55	2.23	319
	Rereading duration	233.64	161.66	35.74	1184.77	2.50	322	178.47	163.79	18.28	1953.87	4.90	319
	Initial fixation count	1.51	0.22	1.15	2.40	1.11	322	1.42	0.22	1.06	2.53	1.59	319
	Total fixation count	2.28	0.59	1.39	5.41	1.75	322	2.01	0.60	1.17	7.23	3.16	319
	Total gaze count	1.54	0.23	1.08	2.61	1.59	322	1.42	0.25	1.08	3.15	2.21	319
	Saccade amplitude	2.37	0.51	1.25	4.79	1.07	322	2.67	0.78	1.34	6.29	1.30	319
	Initial landing position	2.22	0.23	1.49	2.72	− 0.14	322	2.34	0.28	1.47	3.15	− 0.09	319
5	Initial fixation duration	289.93	57.52	172.39	517.24	0.86	301	277.67	57.02	165.84	497.67	0.74	306
	Refixation duration	132.86	79.85	24.83	570.11	1.82	301	105.52	75.04	6.87	631.38	2.05	306
	Rereading duration	204.44	161.85	39.22	1345.22	3.21	301	145.10	124.09	12.38	1307.51	3.69	306
	Initial fixation count	1.46	0.20	1.11	2.41	1.44	301	1.37	0.20	1.04	2.55	1.49	306
	Total fixation count	2.17	0.58	1.37	5.69	2.11	301	1.87	0.49	1.18	5.70	2.28	306
	Total gaze count	1.49	0.24	1.14	3.20	2.21	301	1.37	0.21	1.04	2.96	2.18	306
	Saccade amplitude	2.43	0.57	1.17	5.05	1.02	301	2.77	0.81	1.37	6.29	1.12	306
	Initial landing position	2.23	0.23	1.35	2.84	− 0.22	301	2.37	0.27	1.53	3.16	− 0.11	306
6	Initial fixation duration	273.30	50.29	147.42	503.26	0.98	292	262.12	53.16	171.04	510.18	0.97	290
	Refixation duration	101.97	59.50	21.74	428.39	1.86	292	76.02	53.34	1.93	315.21	1.44	290
	Rereading duration	167.73	105.21	32.63	931.94	2.56	292	119.10	87.21	0.00	618.34	1.70	290
	Initial fixation count	1.38	0.17	1.11	2.31	1.56	292	1.29	0.16	1.01	1.98	1.23	290
	Total fixation count	2.00	0.44	1.27	4.09	1.68	292	1.72	0.40	1.01	3.41	1.23	290
	Total gaze count	1.47	0.19	1.12	2.40	1.19	292	1.34	0.18	1.00	2.09	1.00	290
	Saccade amplitude	2.40	0.57	1.25	4.83	0.96	292	2.99	0.91	1.27	7.80	1.32	290
	Initial landing position	2.27	0.27	1.54	2.87	0.05	292	2.29	0.23	1.70	2.92	− 0.07	290

Duration is in ms. Initial fixation duration = average of fixation duration, Refixation duration = average of refixation duration (in first-pass reading), Rereading duration = average of rereading duration (in first-pass reading), Initial fixation count = average of number of fixations in first-pass reading, Total fixation count = average of total number of fixations on the word, Total gaze count = average of total number of passes (gazes) on the word, Saccade amplitude = average of incoming saccade amplitude (in first-pass reading), Initial landing position = average of initial landing position (in first-pass reading).

**Table 2:** Mean comparisons between oral versus silent reading mode among eye-tracking measures by wave.

Wave	Measure	*t* (*df*)	*p* value
1	Initial fixation duration	− 3.76 (542)	< 0.001
	Refixation duration	6.62 (542)	< 0.001
	Rereading duration	7.68 (542)	< 0.001
	Initial fixation count	7.10 (542)	< 0.001
	Total fixation count	8.41 (542)	< 0.001
	Total gaze count	7.30 (542)	< 0.001
	Saccade amplitude	− 6.85 (542)	< 0.001
	Initial landing position	− 7.93 (542)	< 0.001
2	Initial fixation duration	4.72 (681)	< 0.001
	Refixation duration	5.38 (681)	< 0.001
	Rereading duration	6.19 (681)	< 0.001
	Initial fixation count	5.16 (681)	< 0.001
	Total fixation count	6.76 (681)	< 0.001
	Total gaze count	6.48 (681)	< 0.001
	Saccade amplitude	− 8.64 (681)	< 0.001
	Initial landing position	− 5.18 (681)	< 0.001
3	Initial fixation duration	3.15 (643)	0.002
	Refixation duration	5.54 (643)	< 0.001
	Rereading duration	5.89 (643)	< 0.001
	Initial fixation count	5.23 (643)	< 0.001
	Total fixation count	6.44 (643)	< 0.001
	Total gaze count	6.31 (643)	< 0.001
	Saccade amplitude	− 6.69 (643)	< 0.001
	Initial landing position	− 6.97 (643)	< 0.001
4	Initial fixation duration	2.50 (639)	< 0.001
	Refixation duration	4.44 (639)	< 0.001
	Rereading duration	4.29 (639)	< 0.001
	Initial fixation count	5.25 (639)	< 0.001
	Total fixation count	5.87 (639)	< 0.001
	Total gaze count	5.95 (639)	< 0.001
	Saccade amplitude	− 5.90 (639)	< 0.001
	Initial landing position	− 6.11 (639)	< 0.001
5	Initial fixation duration	2.64 (605)	0.009
	Refixation duration	4.35 (605)	< 0.001
	Rereading duration	5.07 (605)	< 0.001
	Initial fixation count	5.43 (605)	< 0.001
	Total fixation count	6.80 (605)	< 0.001
	Total gaze count	6.82 (605)	< 0.001
	Saccade amplitude	− 5.97 (605)	< 0.001
	Initial landing position	− 6.71 (605)	< 0.001
6	Initial fixation duration	2.61 (580)	0.009
	Refixation duration	5.54 (580)	< 0.001
	Rereading duration	6.07 (580)	< 0.001
	Initial fixation count	6.84 (580)	< 0.001
	Total fixation count	8.04 (580)	< 0.001
	Total gaze count	8.29 (580)	< 0.001
	Saccade amplitude	− 9.28 (580)	< 0.001
	Initial landing position	− 1.29 (580)	0.198

Within each reading mode, substantial developmental changes were observed over time. In the oral reading condition, mean initial fixation duration was 376 ms at the beginning of Grade 1, which decreased to 273 ms at the end of Grade 3. A striking reduction was observed in rereading duration. The mean rereading duration was 632 ms at Wave 1 (fall of Grade 1), which decreased to 376 ms, 311 ms, 234 ms, 204 ms, and 168 ms at Waves 2 to 6, respectively. Similar decreases were observed in the silent reading condition. An opposite pattern was consistently observed for spatial eye-movement measures (saccade amplitude and initial landing position) such that saccade amplitude and initial landing position values were consistently larger in the silent reading mode than in oral reading mode. Beyond the changes in mean values over time, it is notable that standard deviations of temporal eye-movement measures also consistently decreased over time, indicating smaller variation across children in temporal eye-movement measures over time.

The strength of correlations (|*r*|) between eye-movement measures ranged from minimal (0.00) to large (0.96) for both oral and silent reading conditions, and displayed similar patterns across waves (see Table 3).

**Table 3:** Correlations among eye-tracking measures by wave and oral versus silent reading mode.

Wave	Measure	1	2	3	4	5	6	7	8
1	1. Initial fixation duration	–	0.64*	0.42*	0.43*	0.33*	0.14*	− 0.41*	− 0.37*
	2. Refixation duration	0.62*	–	0.66*	0.91*	0.75*	0.40*	− 0.39*	− 0.62*
	3. Rereading duration	0.40*	0.69*	–	0.57*	0.93*	0.90*	− 00.1	− 0.40*
	4. Initial fixation count	0.41*	0.92*	0.58*	–	0.76*	0.38*	− 0.48*	− 0.68*
	5. Total fixation count	0.33*	0.75*	0.96*	0.71*	–	0.86*	− 0.27*	− 0.51*
	6. Total gaze count	0.19*	0.49*	0.94*	0.42*	0.91*	–	− 00.1	− 0.29*
	7. Saccade amplitude	− 0.42*	− 0.36*	00.03	− 0.38*	− 0	0.15*	–	0.59*
	8. Initial landing position	− 0.50*	− 0.68*	− 0.50*	− 0.68*	− 0.56*	− 0.35*	0.41*	–
2	1. Initial fixation duration	–	0.68*	0.47*	0.46*	0.37*	0.19*	− 0.43*	− 0.52*
	2. Refixation duration	0.73*	–	0.67*	0.93*	0.78*	0.42*	− 0.50*	− 0.68*
	3. Rereading duration	0.46*	0.70*	–	0.61*	0.92*	0.90*	− 0.27*	− 0.49*
	4. Initial fixation count	0.50*	0.92*	0.62*	–	0.81*	0.42*	− 0.51*	− 0.66*
	5. Total fixation count	0.36*	0.75*	0.94*	0.76*	–	0.85*	− 0.37*	− 0.58*
	6. Total gaze count	0.16*	0.44*	0.92*	0.42*	0.88*	–	− 0.18*	− 0.34*
	7. Saccade amplitude	− 0.52*	− 0.53*	− 0.16*	− 0.53*	− 0.23*	00.05	–	0.66*
	8. Initial landing position	− 0.48*	− 0.65*	− 0.43*	− 0.64*	− 0.50*	− 0.26*	0.53*	–
3	1. Initial fixation duration	–	0.70*	0.48*	0.46*	0.37*	0.18*	− 0.53*	− 0.53*
	2. Refixation duration	0.74*	–	0.66*	0.92*	0.77*	0.40*	− 0.58*	− 0.68*
	3. Rereading duration	0.49*	0.73*	–	0.57*	0.89*	0.91*	− 0.29*	− 0.48*
	4. Initial fixation count	0.56*	0.94*	0.67*	–	0.81*	0.39*	− 0.56*	− 0.68*
	5. Total fixation count	0.43*	0.80*	0.95*	0.81*	–	0.83*	− 0.39*	− 0.59*
	6. Total gaze count	0.20*	0.47*	0.92*	0.46*	0.87*	–	− 0.14*	− 0.33*
	7. Saccade amplitude	− 0.66*	− 0.65*	− 0.30*	− 0.59*	− 0.33*	− 0	–	0.73*
	8. Initial landing position	− 0.55*	− 0.69*	− 0.49*	− 0.67*	− 00.6	− 0.30*	0.71*	–
4	1. Initial fixation duration	–	0.68*	0.58*	0.48*	0.47*	0.33*	− 0.51*	− 0.51*
	2. Refixation duration	0.64*	–	0.68*	0.94*	0.80*	0.48*	− 0.57*	− 0.65*
	3. Rereading duration	0.42*	0.72*	–	0.60*	0.92*	0.93*	− 0.31*	− 0.50*
	4. Initial fixation count	0.43*	0.94*	0.65*	–	0.81*	0.45*	− 0.55*	− 0.64*
	5. Total fixation count	0.31*	0.79*	0.93*	0.82*	–	0.87*	− 0.39*	− 0.58*
	6. Total gaze count	00.1	0.46*	0.90*	0.46*	0.86*	–	− 0.17*	− 0.38*
	7. Saccade amplitude	− 0.58*	− 0.55*	− 0.24*	− 0.48*	− 0.28*	− 0	–	0.71*
	8. Initial landing position	− 0.35*	− 0.58*	− 0.39*	− 0.59*	− 0.48*	− 0.25*	0.61*	–
5	1. Initial fixation duration	–	0.74*	0.62*	0.57*	0.54*	0.41*	− 0.56*	− 0.58*
	2. Refixation duration	0.69*	–	0.76*	0.96*	0.86*	0.59*	− 0.66*	− 0.69*
	3. Rereading duration	0.48*	0.68*	–	0.69*	0.93*	0.94*	− 0.46*	− 0.53*
	4. Initial fixation count	0.44*	0.92*	0.60*	–	0.87*	0.57*	− 0.65*	− 0.66*
	5. Total fixation count	0.35*	0.77*	0.92*	0.80*	–	0.88*	− 0.54*	− 0.60*
	6. Total gaze count	0.19*	0.45*	0.92*	0.44*	0.87*	–	− 0.34*	− 0.41*
	7. Saccade amplitude	− 0.57*	− 0.62*	− 0.22*	− 0.54*	− 0.29*	0	–	0.72*
	8. Initial landing position	− 0.51*	− 0.67*	− 0.45*	− 0.65*	− 0.53*	− 0.29*	0.66*	–
6	1. Initial fixation duration	–	0.70*	0.54*	0.51*	0.42*	0.30*	− 0.43*	− 0.43*
	2. Refixation duration	0.67*	–	0.71*	0.95*	0.80*	0.56*	− 0.56*	− 0.59*
	3. Rereading duration	0.43*	0.64*	–	0.68*	0.93*	0.94*	− 0.32*	− 0.48*
	4. Initial fixation count	0.42*	0.92*	0.57*	–	0.85*	0.58*	− 0.55*	− 0.59*
	5. Total fixation count	0.29*	0.73*	0.91*	− 00.5	–	0.91*	− 0.40*	− 0.54*
	6. Total gaze count	00.12	0.39*	0.92*	0.39*	0.86*	–	− 0.21*	− 0.41*
	7. Saccade amplitude	− 0.55*	− 0.60*	− 0.21*	− 0.54*	− 0.28*	00.01	–	0.71*
	8. Initial landing position	− 0.40*	− 0.54*	− 0.45*	− 0.54*	− 0.52*	− 0.35*	0.48*	–

Oral reading correlations on the lower diagonal. Silent reading correlations on the upper diagonal.

*p < 0.05.

### Research question 2: variability in eye movements attributable to between-child and between-text differences

The unconditional LMEMs to estimate variance and ICCs were estimated and summarized by wave for the oral reading (Table S1) and silent reading (Table S2) conditions. At each wave, the majority of variance in eye-movement measures in the oral reading condition was attributed to between-student differences: Wave 1 (0.50–0.84), Wave 2 (0.60–0.76), Wave 3 (0.50–0.77), Wave 4 (0.44–0.80), Wave 5 (0.36–0.81), and Wave 6 (0.44–1.00). The student ICCs tended to be smaller for initial landing position (Table S1) where the residual variances ranged from 0.19 to 0.40. The passage-level ICCs by wave were as follows: Wave 1 (0.00–0.13), Wave 2 (0.00–0.02), Wave 3 (0.00–0.13), Wave 4 (0.00–0.22), Wave 5 (0.00–0.36), and Wave 6 (0.00–0.01).

The majority of variance in eye-movement measures in the silent reading condition was also attributed to between-student differences: Wave 1 (0.50–0.80), Wave 2 (0.50–0.84), Wave 3 (0.44–0.79), Wave 4 (0.46–0.80), Wave 5 (0.43–0.82), and Wave 6 (0.50–1.00). Similar to the oral reading condition, the lower student ICCs were for initial landing position (Table S2) where the residual variances ranged from 0.21 to 0.40. The passage-level ICCs by wave were as follows: Wave 1 (0.00–0.10), Wave 2 (0.00–0.13), Wave 3 (0.00–0.11), Wave 4 (0.00–0.23), Wave 5 (0.00–0.36), and Wave 6 (0.00–0.01). The maximum ICC values by wave were consistently associated with initial landing position.

### Research question 3: functional form of growth trajectories of eye movements

Considering the unconditional model ICCs, eye-movement measures across the passages were aggregated for the individual growth curve LMEMs. Only initial landing position had moderate levels of passage ICCs, with the remaining measures showing < 5% due to passage effects. Linear, quadratic, and cubic model fit results are reported in Table S3; random effects associated with the intercept and slope portions of the model are reported in Table 4; and fixed effects are reported in Table 5.

**Table 4:** Random effect coefficients for random effects growth models by oral and silent reading mode.

Measure	Parameter	Oral		Silent	
		Variance	ICC	Variance	ICC
Initial fixation duration	Intercept	5835.23	0.86	5122.76	0.85
	Linear	29.04	< 0.001	28.04	< 0.001
	Quadratic	0.01	< 0.001	0.01	< 0.001
	Cubic	–	–	–	–
	Residual	882.33	0.13	873.74	0.14
Refixation duration	Intercept	33,698.82	0.93	20,440.92	0.90
	Linear	257.67	0.01	183.16	0.01
	Quadratic	0.13	< 0.001	0.10	< 0.001
	Cubic	–	–	–	–
	Residual	2454.96	0.06	1932.47	0.08
Rereading duration	Intercept	70,491.84	0.77	78,980.21	0.86
	Linear	5234.98	0.06	656.42	< 0.001
	Quadratic	7.12	< 0.001	0.37	< 0.001
	Cubic	0.01	< 0.001	–	< 0.001
	Residual	14,173.84	0.16	11,393.97	0.13
Initial fixation count	Intercept	0.10	0.82	0.04	0.66
	Linear	< 0.01	< 0.001	< 0.01	< 0.001
	Quadratic	< 0.01	< 0.001	–	–
	Cubic	–	–	–	–
	Residual	0.02	0.17	0.02	0.33
Total fixation count	Intercept	1.69	0.90	0.61	0.82
	Linear	0.01	0.01	0.01	0.01
	Quadratic	< 0.01	< 0.001	< 0.01	< 0.001
	Cubic	–	–	–	–
	Residual	0.16	0.09	0.12	0.16
Total gaze count	Intercept	0.30	0.90	0.08	0.72
	Linear	< 0.01	< 0.001	< 0.01	< 0.001
	Quadratic	< 0.01	< 0.001	–	–
	Cubic	–	–	–	–
	Residual	0.03	0.09	0.03	0.27
Saccade amplitude	Intercept	0.33	0.83	0.83	0.74
	Linear	< 0.01	< 0.001	0.01	0.01
	Quadratic	< 0.01	< 0.001	–	–
	Cubic	–	–	–	–
	Residual	0.07	0.16	0.28	0.25
Initial landing position	Intercept	0.03	0.50	0.07	0.70
	Linear	< 0.01	< 0.001	0.01	0.10
	Quadratic	< 0.01	< 0.001	< 0.01	< 0.001
	Cubic	–	–	< 0.01	< 0.001
	Residual	0.03	0.49	0.02	0.19

**Table 5:** Fixed effect coefficients for random effects growth models by oral and silent reading mode.

Measure	Parameter	Oral			Silent		
		Est	*SE*	*p*	Est	*SE*	*p*
Initial fixation duration	Intercept	385.06	4.40	< .0001	357.81	4.22	< .0001
	Linear	− 4.81	0.39	< .0001	− 3.94	0.39	< .0001
	Quadratic	0.04	0.01	< .0001	0.03	0.01	0.004
	Cubic	–	–	–	–	–	–
Refixation duration	Intercept	326.65	10.16	< .0001	244.10	8.35	< .0001
	Linear	− 12.80	0.97	< .0001	− 10.59	1.16	< .0001
	Quadratic	0.19	0.02	< .0001	0.32	0.07	< .0001
	Cubic	–	–	–	− 0.0049	0.00	< .0001
Rereading duration	Intercept	633.84	16.75	< .0001	343.57	16.49	< .0001
	Linear	− 45.83	4.48	< .0001	− 12.04	1.70	< .0001
	Quadratic	1.90	0.23	< .0001	0.16	0.04	< .0001
	Cubic	− 0.03	0.01	< .0001	–	–	–
Initial fixation count	Intercept	1.80	0.02	< .0001	1.63	0.01	< .0001
	Linear	− 0.02	0.00	< .0001	− 0.01	0.00	< .0001
	Quadratic	0.0002	0.00	< .0001	–	–	–
	Cubic	–	–	–	–	–	–
Total fixation count	Intercept	3.38	0.07	< .0001	2.56	0.05	< .0001
	Linear	− 0.09	0.01	< .0001	− 0.04	0.01	< .0001
	Quadratic	0.0014	0.00	< .0001	0.0003	0.00	0.015
	Cubic	–	–	–	–	–	–
Total gaze count	Intercept	1.91	0.03	< .0001	1.58	0.02	< .0001
	Linear	− 0.03	0.00	< .0001	− 0.0081	0.00	< .0001
	Quadratic	0.0005	0.00	< .0001	–	–	–
	Cubic	–	–	–	–	–	–
Saccade amplitude	Intercept	1.86	0.03	< .0001	2.33	0.06	< .0001
	Linear	− 0.02	0.01	< .0001	0.0059	0.01	0.369
	Quadratic	0.0043	0.00	< .0001	0.0005	0.00	0.003
	Cubic	− 0.0001	0.00	< .0001	–	–	–
Initial landing position	Intercept	1.66	0.01	< .0001	1.90	0.02	< .0001
	Linear	0.03	0.00	< .0001	− 0.02	0.00	< .0001
	Quadratic	− 0.0003	0.00	< .0001	0.0043	0.00	< .0001
	Cubic	–	–	–	− 0.0001	0.00	< .0001

#### Oral reading

**Figure 1:**
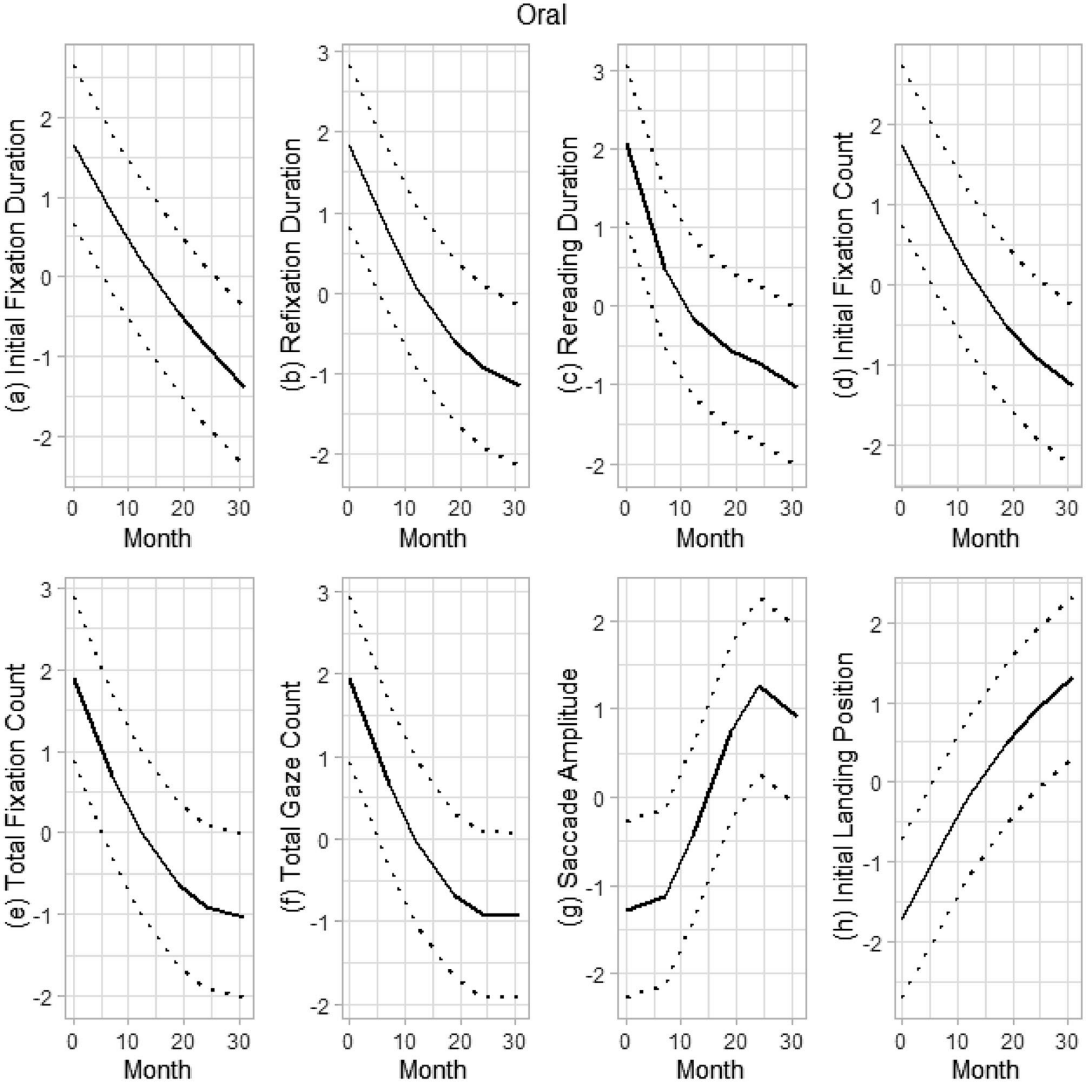
Growth trajectories of eye movements in oral reading.

The quadratic growth model for initial fixation duration showed initial performance of 385.06 (*p* < 0.001) with a negative linear term (− 4.81, *p* < 0.001) and positive quadratic term (0.04, *p* < 0.001) suggesting a decrease in change over time with tapering of growth toward the end of change (see Fig. 1). Refixation duration, rereading duration, initial fixation count, total fixation count, and total gaze count were best fit by quadratic models with decreasing change over time coupled with deceleration, or tapering of growth, at the end of measurement. Saccade amplitude was best fit by a cubic model characterized by initial acceleration in growth followed by deceleration toward the end of measurement (Fig. 1). Initial landing position growth was best fit by a quadratic model with stronger linear change over time that decelerated toward the end of measurement.

#### Silent reading

**Figure 2:**
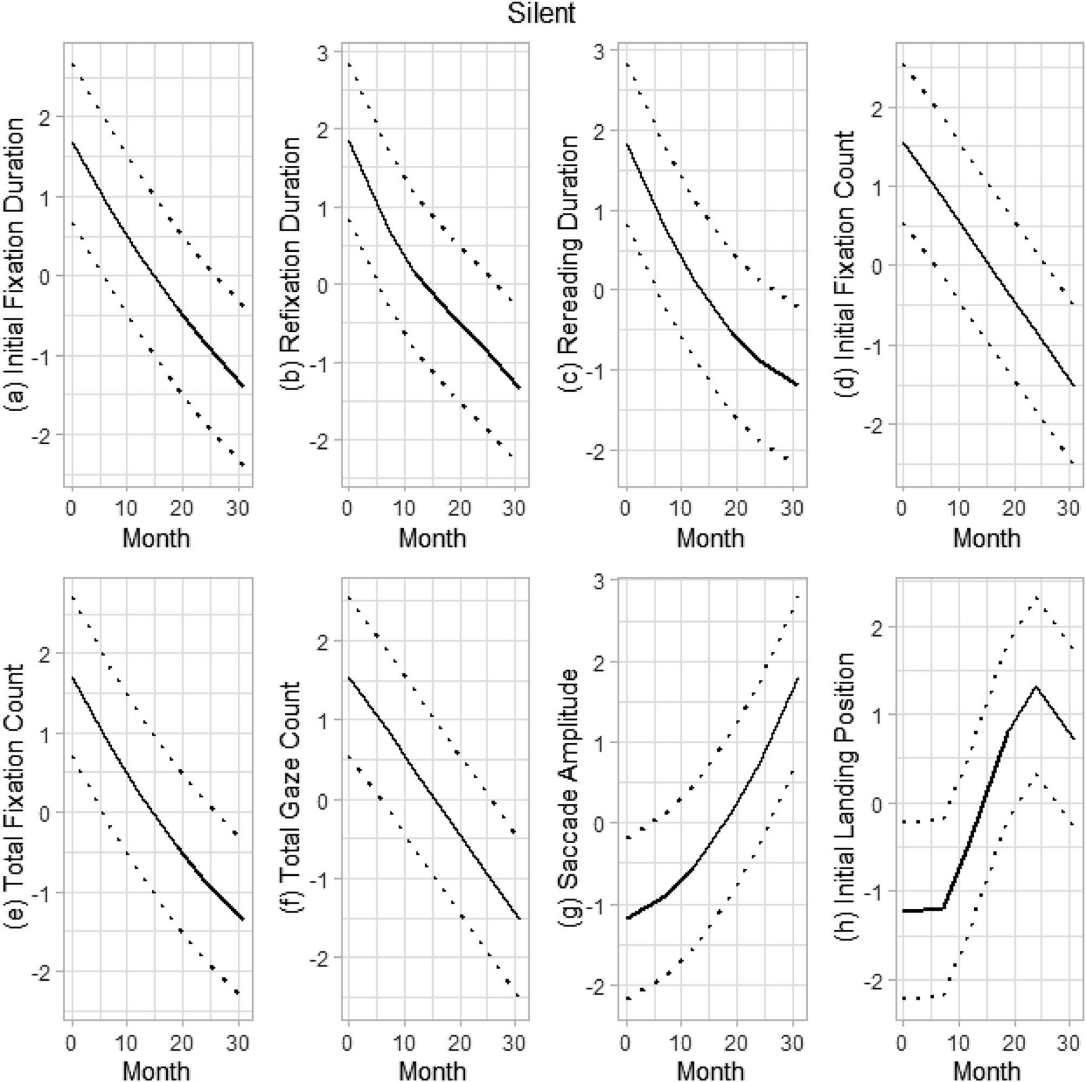
Growth trajectories of eye movements in silent reading.

For the silent reading condition, a linear trend was indicated for initial fixation count and total gaze count, showing a steady decrease in values over time (Fig. 2). A quadratic growth model was the best fitting model for initial fixation duration and rereading duration, indicating a decelerated decrease in values over time. Saccade amplitude was best fit by a quadratic model characterized by accelerated growth across the waves (Fig. 2). Initial landing position growth was best fit by a cubic model with stronger linear change across the middle waves that was punctuated by initial stable values and rapidly decelerated growth towards the end of measurement. Although the graphic representation of change in refixation duration shows elements of quadratic or cubic growth indicated by increased deceleration across waves (Fig. 2), the growth pattern was best captured using a cubic model (Table 5).

## Discussion

In the present study, we examined developmental progression and trajectories of eye movements during text reading in oral and silent reading modes for English-speaking beginning readers, using longitudinal data from Grade 1 to Grade 3. Substantial changes were found in all eye movement parameters during the examined period. In oral reading, the mean initial fixation duration changed from 376 ms at the beginning of Grade 1 to 273 ms at the end of Grade 3. In silent reading, the mean initial fixation duration changed from 351 ms at the beginning of Grade 1 to 262 ms at the end of Grade 3. Similar or greater changes were observed in refixation duration and rereading duration. Fixation and gaze counts also decreased from Grade 1 to Grade 3 in oral and silent reading, albeit changes were not as stark as those in duration.

An opposite pattern was found for initial landing position such that mean values increased from Grade 1 to Grade 3 in oral and silent modes. For example, in oral reading, the mean initial landing position was 1.74 at the beginning of Grade 1 and 2.27 at the end of Grade 3. In other words, children’s initial landing position moved further to the right toward the middle of the word. These results are in line with previous studies^21,22^ and expand our understanding of developmental progression of eye movements using longitudinal data from a large sample of children. These developmental changes are also in line with computational models of reading such as the SWIFT model^5^ and the E-Z reader model^6^ which state that eye movements are mainly driven by word reading and associated orthographic, phonological, and semantic processes, and empirical evidence which showed that students’ reading proficiency is related to their eye movements^17,18,24,43–45^. That is, as children’s reading skill develops, they spend less time and fixate less frequently on each word, and their initial landing position moves further to the right. Although the present study did not examine the development of children’s reading skills (e.g., word reading) and precursors of reading (orthographic, phonological, and semantic processes) together with eye movement parameters, it seems reasonable that changes in eye movements observed in the present study are due to children’s development in reading skills, given well-documented rapid development of reading skills in primary grades and the relation between reading proficiency and eye movements^17,18,24^.

With regard to developmental changes in oral and silent reading modes, overall patterns were similar: decreases in temporal measures and increases in spatial measures. However, differences as a function of reading mode were also found. Children had longer fixation durations and more frequent fixations in oral reading than silent reading whereas mean saccade amplitude and initial landing position values were greater in silent reading than oral reading. These results are convergent with prior work. For example, greater fixation count, longer fixation duration, and smaller amplitude were found in oral compared to silent reading during sentence reading for English-speaking children in elementary grades^22^ and German-speaking adolescents^32^. These differences in oral versus silent reading are likely attributed to the fact that oral reading requires more ongoing processes (see the literature review section above).

A unique aspect of the study is parsing the variability in eye movements to between-child differences and between-text differences. Previous eye-tracking studies have focused on either word features that influence eye movements^8,34,46^ or the relation of a reader characteristic, reading proficiency, to eye movements^17,18,24,44^. To our knowledge, no prior work examined how much variability in eye movements is attributable to between-reader/individual differences and between-text differences. Our findings revealed that for beginning readers in Grades 1 to 3, a large amount of variance in eye movements was attributable to between-student differences: With the exceptions of initial landing position and total gaze count in oral reading in Grade 3, the majority of variance in eye movements was attributable to individual differences (i.e., > 60%). Variance attributable to text differences was mostly minimal. An exception was initial landing position, in which variance ranged from 10 to 36%. Overall, these results indicate that individual differences largely explain developing readers’ eye movements. Individual differences most likely reflect differences in reading proficiency as theoretical models specify reading proficiency as the basis for eye movements^6,7^ and empirical evidence has shown the relation of reading proficiency to eye movements^19,21,24,44^. However, unlike for all the temporal measures of eye movements and saccade amplitude, for initial landing position, it seems that text differences do play a role. This could be related to a higher proportion of longer words in higher grades, where the chance of initially fixating further into a word is simply higher compared to texts with shorter words, independent of reading skill. However, what text features account for differences in initial landing position is beyond the scope of the present study, and therefore, future studies are warranted.

Another unique aspect of this study is investigation of growth trajectories of eye-movement measures during a time when reading develops rapidly. The majority of eye-movement measures had nonlinear growth trajectories where fast development was followed by a slowdown near the end of Grade 3 (see Figs. 1 and 2). In oral and silent reading, initial fixation duration, rereading duration, and total fixation count showed a rapid decrease followed by tapering of growth toward the end of Grade 3. Some differences between oral and silent reading were also observed. For example, saccade amplitude in oral reading grew at a rapid rate followed by a decrease in rate at the end of Grade 3 (a cubic growth pattern) whereas in silent reading, saccade amplitude had faster growth over time (a quadratic growth pattern). An opposite pattern was found for the initial landing position. In addition, initial fixation count and total gaze count in silent reading had a linear growth trajectory while these had a quadratic growth pattern in oral reading.

The growth patterns of fixation duration and fixation count in the present study are divergent from what was reported in an earlier study with an Italian student, which found a rapid decrease in fixation duration and fixation count in Grade 1 followed by slow changes in Grades 2 through 5^29^. These different patterns likely reflect differences in orthographic depth. English has an opaque orthography where grapheme-phoneme correspondences are highly inconsistent, which prolongs development of word reading skills compared to transparent orthographies such as Italian^47^. Therefore, eye-movement measures in deep orthographies will not show as rapid changes during a relatively short period of initial years of schooling as those reported in Italian. It should be noted, however, that the study in Italian was conducted with a single student, and therefore, future longitudinal studies with a larger sample of children learning to read in transparent orthographies are needed.

## Limitations, future directions, and conclusion

In the present study, we examined eye movements for beginning readers from Grade 1 to Grade 3. We found rapid changes in eye-movement measures using age-appropriate reading materials, which allowed us to examine reader and text-based influences throughout this critical period. One could argue that using identical texts in all waves would provide better measures of growth-related individual differences. We decided against this approach in the present study, as using identical texts would open the possibility to over- or underestimate developmental progression if texts are too difficult in the beginning or too easy towards the end of the assessment period.

The current work can be extended in several directions. First, future work can shed light on how growth trajectories of eye movement parameters are related with precursors of reading skills such as rapid automatized naming (RAN)^20,23^, phonological awareness and orthographic awareness, and reading proficiency such as word reading and reading comprehension^17,18,24^. Another direction is examining word and text features for texts used in the present study, and their relations to growth trajectories of eye movement parameters. Furthermore, future longitudinal work with children in upper elementary grades and secondary school is needed to illuminate growth trajectories beyond the beginning phase of reading development. Lastly, comparative work in languages that differ in orthographic depth can reveal similarities and uniqueness in the development of eye movements as a function of orthographic characteristics.

## Supplementary Information

Supplementary Information.

## Data Availability

The datasets generated and/or analyzed during the current study are not publicly available but are available from the corresponding author on reasonable request.
