# Supplementary material for: Developmental trajectories of eye movements in oral and silent reading for beginning readers: a longitudinal investigation
Source: Sci Rep. 2022 Nov 4;12:18708. doi: 10.1038/s41598-022-23420-5 (PMC9636221; doi:10.1038/s41598-022-23420-5)
Supplement: Supplementary file 1 — Supplementary Information. [file 41598_2022_23420_MOESM1_ESM.docx]

**Table S1**

*Oral reading variance and intraclass correlations by measure and wave*

|  |  | Variance | | | | Intraclass Correlation | | | |
| --- | --- | --- | --- | --- | --- | --- | --- | --- | --- |
| Wave | Measure | School | Student | Passage | Residual | School | Student | Passage | Residual |
| 1 | Initial Fixation duration | 87.67 | 5718.90 | 66.48 | 1249.63 | 0.01 | 0.80 | 0.01 | 0.18 |
|  | Refixation Duration | 5917.68 | 31586.24 | 13.85 | 8962.94 | 0.13 | 0.68 | 0.00 | 0.19 |
|  | Rereading Duration | 36191.50 | 228367.20 | 622.30 | 31081.70 | 0.12 | 0.77 | 0.00 | 0.11 |
|  | Initial Fixation Count | 0.02 | 0.09 | 0.00 | 0.03 | 0.14 | 0.64 | 0.00 | 0.22 |
|  | Total Fixation Count | 0.30 | 1.59 | 0.00 | 0.20 | 0.14 | 0.76 | 0.00 | 0.10 |
|  | Total Gaze Count | 0.03 | 0.31 | 0.00 | 0.06 | 0.08 | 0.76 | 0.00 | 0.16 |
|  | Saccade Amplitude | 0.00 | 0.37 | 0.01 | 0.06 | 0.00 | 0.84 | 0.02 | 0.14 |
|  | Initial Landing Position | 0.00 | 0.04 | 0.01 | 0.03 | 0.00 | 0.50 | 0.13 | 0.37 |
| 2 | Initial Fixation duration | 525.50 | 5025.80 | 62.10 | 864.70 | 0.08 | 0.76 | 0.01 | 0.15 |
|  | Refixation Duration | 2100.60 | 13544.40 | 470.70 | 2846.40 | 0.11 | 0.71 | 0.02 | 0.16 |
|  | Rereading Duration | 8155.10 | 64216.50 | 920.40 | 11359.40 | 0.10 | 0.76 | 0.01 | 0.13 |
|  | Initial Fixation Count | 0.01 | 0.06 | 0.00 | 0.02 | 0.11 | 0.67 | 0.00 | 0.22 |
|  | Total Fixation Count | 0.07 | 0.61 | 0.01 | 0.11 | 0.09 | 0.76 | 0.01 | 0.14 |
|  | Total Gaze Count | 0.01 | 0.11 | 0.00 | 0.03 | 0.07 | 0.73 | 0.00 | 0.20 |
|  | Saccade Amplitude | 0.02 | 0.15 | 0.00 | 0.04 | 0.10 | 0.71 | 0.00 | 0.19 |
|  | Initial Landing Position | 0.00 | 0.03 | 0.00 | 0.02 | 0.00 | 0.60 | 0.00 | 0.40 |
| 3 | Initial Fixation duration | 743.00 | 4173.80 | 49.40 | 551.20 | 0.13 | 0.76 | 0.01 | 0.10 |
|  | Refixation Duration | 2740.66 | 10284.76 | 44.24 | 1824.23 | 0.18 | 0.69 | 0.00 | 0.13 |
|  | Rereading Duration | 6859.00 | 51231.00 | 0.00 | 12201.00 | 0.10 | 0.73 | 0.00 | 0.17 |
|  | Initial Fixation Count | 0.01 | 0.05 | 0.00 | 0.01 | 0.14 | 0.71 | 0.00 | 0.15 |
|  | Total Fixation Count | 0.09 | 0.65 | 0.00 | 0.10 | 0.11 | 0.77 | 0.00 | 0.12 |
|  | Total Gaze Count | 0.01 | 0.10 | 0.00 | 0.03 | 0.07 | 0.71 | 0.00 | 0.22 |
|  | Saccade Amplitude | 0.04 | 0.24 | 0.01 | 0.02 | 0.13 | 0.77 | 0.03 | 0.07 |
|  | Initial Landing Position | 0.00 | 0.04 | 0.01 | 0.03 | 0.00 | 0.50 | 0.13 | 0.37 |
| 4 | Initial Fixation duration | 282.60 | 2901.75 | 4.51 | 417.27 | 0.08 | 0.80 | 0.00 | 0.12 |
|  | Refixation Duration | 1284.40 | 6616.70 | 348.10 | 1203.60 | 0.14 | 0.70 | 0.04 | 0.12 |
|  | Rereading Duration | 2286.40 | 22772.20 | 572.40 | 4485.90 | 0.08 | 0.76 | 0.02 | 0.14 |
|  | Initial Fixation Count | 0.01 | 0.04 | 0.00 | 0.01 | 0.17 | 0.67 | 0.00 | 0.16 |
|  | Total Fixation Count | 0.04 | 0.29 | 0.02 | 0.06 | 0.10 | 0.70 | 0.05 | 0.15 |
|  | Total Gaze Count | 0.00 | 0.05 | 0.00 | 0.02 | 0.00 | 0.71 | 0.00 | 0.29 |
|  | Saccade Amplitude | 0.02 | 0.23 | 0.01 | 0.03 | 0.07 | 0.79 | 0.03 | 0.11 |
|  | Initial Landing Position | 0.00 | 0.04 | 0.02 | 0.03 | 0.00 | 0.44 | 0.22 | 0.34 |
| 5 | Initial Fixation duration | 542.00 | 2750.00 | 0.00 | 295.10 | 0.15 | 0.77 | 0.00 | 0.08 |
|  | Refixation Duration | 1031.38 | 5211.84 | 98.96 | 678.08 | 0.15 | 0.74 | 0.01 | 0.10 |
|  | Rereading Duration | 2125.58 | 22973.12 | 4.62 | 4152.27 | 0.07 | 0.79 | 0.00 | 0.14 |
|  | Initial Fixation Count | 0.01 | 0.03 | 0.00 | 0.01 | 0.20 | 0.60 | 0.00 | 0.20 |
|  | Total Fixation Count | 0.03 | 0.30 | 0.00 | 0.04 | 0.08 | 0.81 | 0.00 | 0.11 |
|  | Total Gaze Count | 0.00 | 0.01 | 0.00 | 0.01 | 0.00 | 0.50 | 0.00 | 0.50 |
|  | Saccade Amplitude | 0.05 | 0.28 | 0.01 | 0.02 | 0.14 | 0.78 | 0.03 | 0.05 |
|  | Initial Landing Position | 0.01 | 0.04 | 0.04 | 0.02 | 0.09 | 0.36 | 0.36 | 0.19 |
| 6 | Initial Fixation duration | 364.68 | 2153.42 | 2.86 | 218.08 | 0.13 | 0.79 | 0.00 | 0.08 |
|  | Refixation Duration | 530.38 | 2939.74 | 22.57 | 452.44 | 0.13 | 0.75 | 0.01 | 0.11 |
|  | Rereading Duration | 831.47 | 9689.30 | 91.31 | 2198.90 | 0.06 | 0.76 | 0.01 | 0.17 |
|  | Initial Fixation Count | 0.00 | 0.02 | 0.00 | 0.00 | 0.00 | 1.00 | 0.00 | 0.00 |
|  | Total Fixation Count | 0.02 | 0.17 | 0.00 | 0.03 | 0.09 | 0.77 | 0.00 | 0.14 |
|  | Total Gaze Count | 0.00 | 0.03 | 0.00 | 0.01 | 0.00 | 0.75 | 0.00 | 0.25 |
|  | Saccade Amplitude | 0.04 | 0.28 | 0.00 | 0.02 | 0.12 | 0.82 | 0.00 | 0.06 |
|  | Initial Landing Position | 0.03 | 0.04 | 0.00 | 0.02 | 0.33 | 0.44 | 0.00 | 0.23 |

**Table S2**

*Silent reading variance and intraclass correlations by measure and wave*

|  |  | Variance | | | | Intraclass Correlation | | | |
| --- | --- | --- | --- | --- | --- | --- | --- | --- | --- |
| Wave | Measure | School | Student | Passage | Residual | School | Student | Passage | Residual |
| 1 | Initial Fixation duration | 267.42 | 5043.46 | 7.67 | 1117.71 | 0.04 | 0.78 | 0.00 | 0.18 |
|  | Refixation Duration | 1498.61 | 17901.34 | 20.75 | 5108.36 | 0.06 | 0.73 | 0.00 | 0.21 |
|  | Rereading Duration | 6907.70 | 80872.10 | 656.80 | 19956.40 | 0.06 | 0.74 | 0.01 | 0.19 |
|  | Initial Fixation Count | 0.00 | 0.07 | 0.00 | 0.02 | 0.00 | 0.78 | 0.00 | 0.22 |
|  | Total Fixation Count | 0.05 | 0.68 | 0.01 | 0.14 | 0.06 | 0.77 | 0.01 | 0.16 |
|  | Total Gaze Count | 0.01 | 0.12 | 0.00 | 0.04 | 0.06 | 0.71 | 0.00 | 0.23 |
|  | Saccade Amplitude | 0.03 | 0.84 | 0.02 | 0.16 | 0.03 | 0.80 | 0.02 | 0.15 |
|  | Initial Landing Position | 0.00 | 0.05 | 0.01 | 0.04 | 0.00 | 0.50 | 0.10 | 0.40 |
| 2 | Initial Fixation duration | 163.93 | 4287.87 | 37.72 | 860.79 | 0.03 | 0.80 | 0.01 | 0.16 |
|  | Refixation Duration | 811.70 | 9986.50 | 775.70 | 2101.30 | 0.06 | 0.73 | 0.06 | 0.15 |
|  | Rereading Duration | 1319.00 | 39030.00 | 0.00 | 10960.00 | 0.03 | 0.76 | 0.00 | 0.21 |
|  | Initial Fixation Count | 0.01 | 0.05 | 0.01 | 0.01 | 0.13 | 0.63 | 0.13 | 0.11 |
|  | Total Fixation Count | 0.02 | 0.40 | 0.00 | 0.09 | 0.04 | 0.78 | 0.00 | 0.18 |
|  | Total Gaze Count | 0.00 | 0.07 | 0.00 | 0.03 | 0.00 | 0.70 | 0.00 | 0.30 |
|  | Saccade Amplitude | 0.01 | 0.65 | 0.00 | 0.11 | 0.01 | 0.84 | 0.00 | 0.15 |
|  | Initial Landing Position | 0.00 | 0.04 | 0.01 | 0.03 | 0.00 | 0.50 | 0.13 | 0.37 |
| 3 | Initial Fixation duration | 628.20 | 3580.33 | 11.54 | 623.26 | 0.13 | 0.74 | 0.00 | 0.13 |
|  | Refixation Duration | 1411.10 | 6025.20 | 19.30 | 1567.40 | 0.16 | 0.67 | 0.00 | 0.17 |
|  | Rereading Duration | 2475.00 | 26055.00 | 0.00 | 5105.00 | 0.07 | 0.77 | 0.00 | 0.16 |
|  | Initial Fixation Count | 0.01 | 0.04 | 0.00 | 0.01 | 0.17 | 0.67 | 0.00 | 0.16 |
|  | Total Fixation Count | 0.04 | 0.28 | 0.00 | 0.05 | 0.11 | 0.76 | 0.00 | 0.13 |
|  | Total Gaze Count | 0.00 | 0.06 | 0.00 | 0.02 | 0.00 | 0.75 | 0.00 | 0.25 |
|  | Saccade Amplitude | 0.03 | 0.60 | 0.01 | 0.12 | 0.04 | 0.79 | 0.01 | 0.16 |
|  | Initial Landing Position | 0.01 | 0.04 | 0.01 | 0.03 | 0.11 | 0.44 | 0.11 | 0.34 |
| 4 | Initial Fixation duration | 277.58 | 2919.52 | 2.54 | 442.83 | 0.08 | 0.80 | 0.00 | 0.12 |
|  | Refixation Duration | 940.30 | 6110.80 | 178.30 | 1204.40 | 0.11 | 0.72 | 0.02 | 0.15 |
|  | Rereading Duration | 662.80 | 24615.20 | 294.20 | 5047.30 | 0.02 | 0.80 | 0.01 | 0.17 |
|  | Initial Fixation Count | 0.01 | 0.04 | 0.00 | 0.01 | 0.17 | 0.67 | 0.00 | 0.16 |
|  | Total Fixation Count | 0.02 | 0.32 | 0.01 | 0.06 | 0.05 | 0.78 | 0.02 | 0.15 |
|  | Total Gaze Count | 0.00 | 0.05 | 0.00 | 0.02 | 0.00 | 0.71 | 0.00 | 0.29 |
|  | Saccade Amplitude | 0.04 | 0.55 | 0.01 | 0.08 | 0.06 | 0.81 | 0.01 | 0.12 |
|  | Initial Landing Position | 0.00 | 0.06 | 0.03 | 0.04 | 0.00 | 0.46 | 0.23 | 0.31 |
| 5 | Initial Fixation duration | 591.00 | 2652.40 | 5.27 | 272.09 | 0.17 | 0.75 | 0.00 | 0.08 |
|  | Refixation Duration | 984.13 | 4530.07 | 42.24 | 674.78 | 0.16 | 0.73 | 0.01 | 0.10 |
|  | Rereading Duration | 955.40 | 13874.40 | 0.00 | 2322.20 | 0.06 | 0.81 | 0.00 | 0.13 |
|  | Initial Fixation Count | 0.01 | 0.03 | 0.00 | 0.01 | 0.20 | 0.60 | 0.00 | 0.20 |
|  | Total Fixation Count | 0.02 | 0.21 | 0.00 | 0.03 | 0.08 | 0.81 | 0.00 | 0.11 |
|  | Total Gaze Count | 0.00 | 0.04 | 0.00 | 0.01 | 0.00 | 0.80 | 0.00 | 0.20 |
|  | Saccade Amplitude | 0.05 | 0.59 | 0.01 | 0.07 | 0.07 | 0.82 | 0.01 | 0.10 |
|  | Initial Landing Position | 0.00 | 0.06 | 0.05 | 0.03 | 0.00 | 0.43 | 0.36 | 0.21 |
| 6 | Initial Fixation duration | 446.40 | 2324.16 | 2.88 | 316.67 | 0.14 | 0.75 | 0.00 | 0.11 |
|  | Refixation Duration | 422.20 | 2303.60 | 23.60 | 535.60 | 0.13 | 0.70 | 0.01 | 0.16 |
|  | Rereading Duration | 580.56 | 6510.93 | 73.15 | 1981.98 | 0.06 | 0.71 | 0.01 | 0.22 |
|  | Initial Fixation Count | 0.00 | 0.02 | 0.00 | 0.00 | 0.00 | 1.00 | 0.00 | 0.00 |
|  | Total Fixation Count | 0.02 | 0.13 | 0.00 | 0.03 | 0.12 | 0.69 | 0.00 | 0.19 |
|  | Total Gaze Count | 0.00 | 0.03 | 0.00 | 0.01 | 0.00 | 0.75 | 0.00 | 0.25 |
|  | Saccade Amplitude | 0.12 | 0.70 | 0.00 | 0.08 | 0.01 | 0.78 | 0.00 | 0.21 |
|  | Initial Landing Position | 0.01 | 0.04 | 0.00 | 0.03 | 0.13 | 0.50 | 0.00 | 0.37 |

Table S3

*Likelihood ratio tests for random effects growth models by oral and silent reading*

|  |  | Oral | | | |  |  | Silent | | | | |  |  |
| --- | --- | --- | --- | --- | --- | --- | --- | --- | --- | --- | --- | --- | --- | --- |
| Measure | Model | χ^2^ | | df | *p* | AIC | BIC | χ^2^ | df | | | *p* | AIC | BIC |
| Initial Fixation Duration | Linear | -- | -- | | -- | 19610 | 19643 | -- | | -- | -- | | 19251 | 19284 |
|  | Quadratic | 157.19 | 4 | | <.001 | 19460 | 19516 | 135.41 | | 4 | <.001 | | 19124 | 19179 |
|  | Cubic | 0 | 5 | | 1.00 | 21760 | 21843 | 0 | | 5 | 1.00 | | 21624 | 21707 |
| Refixation Duration | Linear | -- | -- | | -- | 19610 | 19643 | -- | | -- | -- | | 21158 | 21191 |
|  | Quadratic | 641.56 | 4 | | <.001 | 21570 | 21625 | 434.48 | | 4 | <.001 | | 20732 | 20787 |
|  | Cubic | 0 | 5 | | 1.00 | 23954 | 24037 | 0 | | 5 | 1.00 | | 22998 | 23080 |
| Rereading Duration | Linear | -- | -- | | -- | 25432 | 25465 | -- | | -- | -- | | 23906 | 23939 |
|  | Quadratic | 676.87 | 4 | | <.001 | 24763 | 24819 | 289.97 | | 4 | <.001 | | 23624 | 23679 |
|  | Cubic | 0 | 5 | | 1.00 | 27194 | 27277 | 0 | | 5 | 1.00 | | 26902 | 26985 |
| Initial Fixation Count | Linear | -- | -- | | -- | -748.71 | -715.52 | -- | | -- | -- | | -998.86 | -965.77 |
|  | Quadratic | 248.84 | 4 | | <.001 | -989.55 | -934.24 | 178.97 | | 4 | <.001 | | -1169.84 | -1114.68 |
|  | Cubic | 0 | 5 | | 1.00 | 1552.99 | 1635.96 | 0 | | 5 | 1.00 | | 1289.8 | 1372.5 |
| Total Fixation Count | Linear | -- | -- | | -- | 3753.2 | 3786.3 | -- | | -- | -- | | 2751.3 | 2784.4 |
|  | Quadratic | 439.67 | 4 | | <.001 | 3321.5 | 3376.8 | 220.31 | | 4 | <.001 | | 2539.0 | 2594.1 |
|  | Cubic | 0 | 5 | | 1.00 | 6086.2 | 6169.2 | 0 | | 5 | 1.00 | | 5679.5 | 5762.3 |
| Total Gaze Count | Linear | -- | -- | | -- | 526.30 | 559.49 | -- | | -- | -- | | -227.74 | -194.64 |
|  | Quadratic | 404.81 | 4 | | <.001 | 129.49 | 184.80 | 124.27 | | 4 | <.001 | | -344.01 | -288.85 |
|  | Cubic | 0 | 5 | | 1.00 | 3561.8 | 3644.7 | 0 | | 5 | 1.00 | | 2108.40 | 2191.14 |
| Saccade Amplitude | Linear | -- | -- | | -- | 2293.6 | 2326.8 | -- | | -- | -- | | 4118.2 | 4151.3 |
|  | Quadratic | 177.47 | 4 | | <.001 | 2124.1 | 2179.4 | 71.46 | | 4 | <.001 | | 4054.7 | 4109.9 |
|  | Cubic | 0 | 5 | | 1.00 | 3826.6 | 3909.6 | 0 | | 5 | 1.00 | | 5525.8 | 5608.5 |
| Initial Landing Position | Linear | -- | -- | | -- | -440.40 | -407.21 | -- | | -- | -- | | 161.939 | 195.03 |
|  | Quadratic | 57.55 | 4 | | <.001 | -489.94 | -434.63 | 108.88 | | 4 | <.001 | | 61.062 | 116.22 |
|  | Cubic | 0 | 5 | | 1.00 | 2211.87 | 2294.83 | 0 | | 5 | 1.00 | | 102.249 | 184.99 |
